# Editorial: Debates in cardiovascular pharmacology and drug discovery: 2022

**DOI:** 10.3389/fcvm.2023.1304680

**Published:** 2023-10-18

**Authors:** Keman Xu, Fatma Saaoud, Ying Shao, Yifan Lu, Xiaohua Jiang, Sheng Wu, Jianxin Sun, Filipe Fernades Conti, Laisel Martinez, Roberto Vazquez-Padron, Hong Wang, Xiaofeng Yang

**Affiliations:** ^1^ Lemore Center for Integrated Lymphatics and Vascular Research, Lewis Katz School of Medicine at Temple University, Philadelphia, PA, United States; ^2^ Centers of Metabolic Disease Research and Thrombosis Research Center, Departments of Cardiovascular Sciences, Lewis Katz School of Medicine at Temple University, Philadelphia, PA, United States; ^3^ Center for Translational Medicine, Vascular Biology and Therapeutics Program, Department of Medicine, Thomas Jefferson University, Philadelphia, PA, United States; ^4^ DeWitt Daughtry Family Department of Surgery, Leonard M. Miller School of Medicine, University of Miami, Miami, FL, United States

**Keywords:** metabolomics, cardiovascular diseases, cardiovascular injuries, inflammation, trained immunity

**Editorial on the Research Topic** Debates in cardiovascular pharmacology and drug discovery: 2022

## Introduction

Cardiovascular diseases (CVDs) are the leading cause of death worldwide. High-throughput metabolomics analysis is a powerful tool in characterizing metabolomic reprogramming related to various diseases in biomedical fields, uncovering the underlying novel metabolic mechanisms, and identifying diagnostic biomarkers (1, 2). Yet, our understanding of the metabolite-based danger-associated molecular patterns (DAMPs) (3, 4) amplified inflammation during CVDs (5) is not fully elucidated. Our Research Topic: Debates in Cardiovascular Pharmacology and Drug Discovery: 2022 at the Frontiers in Cardiovascular Medicine highlighted five papers. Those highlights encompass original research papers and reviews and provide a comprehensive view of drugs and metabolic mechanisms associated with CVDs. In 2023, we will continue to maintain an exceptional platform that fosters the exchange of groundbreaking insights among cardiologists, translational cardiovascular researchers, and experts in cardiovascular pharmacology and drug discovery.

## Metabolic reprogramming and trained immunity/inflammation constitute a significant risk factor for the development of cardiovascular diseases

CVDs, for the most part, originate from disturbances in cardiovascular metabolism (6–9). Metabolites are the dynamic feedback of metabolism in cells, tissues, or organisms. They are uniquely positioned in the ‘omics’ hierarchy, representing the terminal products of processes originating from other “upstream levels” such as the genome, epigenome, transcriptome, and proteome. Metabolomics, a cutting-edge technology, has become a valuable tool for studying CVDs by revealing changes in metabolism. Comparing metabolite variations in the presence of pathophysiological conditions offers additional insights into the metabolomic mechanisms underlying the pathogenesis of diseases (2).

The development of CVDs is closely tied to inflammation pathologies. Nevertheless, the precise mechanisms through which metabolic reprogramming promotes inflammation in the context of CVD remain elusive. Earlier publications have indicated that persistent inflammatory metabolic reprogramming of innate immune cells and their bone marrow precursors plays a pivotal role in the onset and progression of CVDs (10). This metabolic reprogramming is associated with the concept of trained immunity (also termed innate immune memory) (2, 11). To be more specific, trained immunity is achieved through metabolic alterations within innate immune cells in response to stimuli. This process involves histone modifications such as methylation and acetylation, resulting in enduring epigenetic changes in inflammation-associated gene promoters and activating four metabolic pathways, including glycolysis, acetyl coenzyme A (acetyl-CoA) generation, mevalonate-cholesterol synthesis, and glutaminolysis (5). Innate immune cells that have undergone this “training” and developed stimuli-non-specific memories exhibit more robust responses when faced with the potential for re-stimulation (12).

Nevertheless, a significant question that remains is: how do different CVDs simultaneously and uniformly reprogram all four metabolic pathways? A recent publication has revealed that during early hyperlipidemia-induced atherosclerosis, three distinct types of trained immunity coexist. Each of these trained immunity types is characterized by specific mechanisms, including acetyl-CoA-cholesterol-driven histone acetylation dependence, S-adenosylhomocysteine (SAH) hypomethylation-triggered histone demethylation dependence, and glycolysis-independent metabolic pathways (2). These concurrent forms of trained immunity collectively play a pivotal role in exacerbating inflammation (13–20) and driving the progression of atherosclerosis (2, 21). In addition, this paper clearly showed that metabolomic reprogramming exhibits pronounced specificity at the tissue and subcellular organelle levels (22, 23). It is noteworthy that a significant number of upregulated metabolites are prominently expressed within the mitochondria of the heart. This underscores the elevated energy demand (24) associated with the initial stages of atherosclerosis in the cardiac tissue (2).

## Five highlights of our research topic relate to therapeutic studies in metabolic reprograming, inflammation, and trained immunity

Metabolomics plays a crucial role in delineating pathophysiological responses and identifying markers for potential drug candidates. As we mentioned above, mitochondria (25) play a major role in the process of atherosclerosis, and a similar result was also reported by Xiang et al. in myocardial infarction (MI). Xiang’s group investigated a traditional Chinese medicine called Modified Linggui Zhugan Decoction (MLZD), which protects against MI by improving mitochondrial functions and reversing ventricular remodeling (26). In addition, Wu et al. examined the association of estimated pulse wave velocity with all causes and cardiovascular mortality in patients with diabetes (27). Jiang et al. comprehensively summarized the signaling pathway of cross-interference between autophagy and different types of CVDs (28). Our research topic not only emphasizes the investigation of underlying mechanisms but also provides comprehensive reviews of cardiovascular damage resulting from drug toxicities. Liu et al. conducted a review that centered on targeted drug toxicity affecting the heart in the context of breast cancer (29), whereas Gona et al. delved into the subject of cardiovascular toxicity in relation to digoxin (30).

We listed these five key highlights in Table 1, and we are confident that the articles within our research topic offer fresh perspectives on metabolomic reprogramming associated with CVDs. This reprogramming presents therapeutic targets and drug toxicities for treating a variety of disease conditions, including as metabolic CVDs, diabetes, and breast cancer. It also enables conceptual innovation of trained immunity and disease progression.

**Table 1:** Five highlights of our research topic.

Paper types	Title	PMID
Original research	Modified Linggui Zhugan Decoction protects against ventricular remodeling through ameliorating mitochondrial damage in post-myocardial infarction rats	36704451
Original research	Estimated pulse wave velocity is associated with all-cause mortality and cardiovascular mortality among adults with diabetes	37139122
Review	Cardiotoxicity from neoadjuvant targeted treatment for breast cancer prior to surgery	36910540
Review	The role of autophagy in cardiovascular disease: Cross-interference of signaling pathways and underlying therapeutic targets	37063954
Policy and practice reviews	Review: Failure of current digoxin monitoring for toxicity: new monitoring recommendations to maintain therapeutic levels for efficacy	37465455
